# Potential High Arbovirus Exposure in INDOPACOM During U.S. Service Member Deployment or Exercises in Papua New Guinea

**Published:** 2025-08-20

**Authors:** Joanne Kizu, Melissa Graham, Rhys Izuagbe, Brady McPherson, Peter Kaminiel, Wenjun Liu

**Affiliations:** Australian Defense Force Malaria and Infectious Disease Institute, Gallipoli Barracks, Enoggera, Queensland: CAPT Kizu, CAPT Graham, LT Izuagbe, LCOL McPherson, Dr. Liu; Queensland Institute of Medical Research, Berghofer Medical Research Institute, Brisbane, Australia: Ms. Graham; Health Services, Papua New Guinea Defence Force, Port Moresby: Dr. Kaminiel

## Abstract

Arboviruses pose a significant health threat to U.S. military personnel deployed in the U.S. Indo-Pacific Command (INDOPACOM) region. In 2023 we conducted a sero-epidemiological study to determine the arboviruses circulating in 185 Papua New Guinea military personnel (PNGMP), using the neutralizing antibody (NAb) assay. Overall, sero-positivity rates among the 185 PNGMP tested were: anti-Zika virus (ZIKV), 87% (n=161); anti-Japanese encephalitis virus (JEV), 62.2% (n=115); anti-Ross River virus (RRV), 44.3% (n=82); anti-Murray Valley encephalitis virus (MVEV), 39.5% (n=73); anti-chikungunya virus (CHIKV), 33.5% (n=62); anti-Barmah Forest virus (BFV), 10.8% (n=20); and anti-West Nile virus (WNV), 5.9% (n=11). The monotypic NAb sero-positivity rates for dengue virus (DENV) serotypes were: anti-DENV-1 94.6% (n=175), anti-DENV-2 93% (n=172), anti-DENV-3 95.1% (n=176), and anti-DENV-4 31.4% (n=57). These findings indicate that the majority of PNGMP had prior exposure to DENV and ZIKV, with a notable proportion exposed to CHIKV, RRV, JEV, and MVEV, and lower levels of exposure to BFV and WNV. Low or moderate prior exposure may leave individual PNGMP immunologically naïve and more susceptible to infection and disease upon first exposure. Furthermore, secondary DENV infections with a different serotype can increase risk of severe disease due to immune enhancement mechanisms such as antibodydependent enhancement. Understanding these exposure patterns is crucial for assessing population risk and informing surveillance and prevention strategies. U.S. soldiers exercising or deploying to Papua New Guinea should adhere to strict preventive measures for minimizing mosquito bites and reducing their risk of arboviral infections.

Arboviral diseases, transmitted by arthropods such as mosquitoes and ticks, are a significant health threat to deployed U.S. military personnel and the U.S. Military Health System (MHS). Arboviruses pose continual risk, and continue to emerge and challenge force health protection. With new outbreaks and evolving vector ecology across the Indo-Pacific region, risk of both endemic and emerging arboviruses remains. Updated sero-surveillance helps to track trends, guide prevention, and strengthen operational readiness.

The U.S. military has a long history of combating arboviral diseases, particularly dengue virus (DENV), Zika virus (ZIKV), and chikungunya virus (CHIKV), with DENV the most prevalent. ^1^ DENV infections have remained a persistent operational threat to the U.S. military since the Spanish–American War. ^1^ The first isolation of DENV was achieved by Dr. Albert Sabin in 1944, derived from a U.S. soldier who experienced acute illness while deployed to Papua New Guinea. ^2^ The original New Guinea C strain of DENV, now known to be a DENV-2 serotype, was followed in the same year by additional isolation, of DENV-1, from another sick soldier stationed in Papua New Guinea. ^3^

**What are the new findings?**

To our knowledge, this study provides the first comprehensive examination of arbovirus sero-positivity rates in Papua New Guinea military personnel (PNGMP) following the COVID-19 pandemic. After examining sero-positivity of 11 arboviruses, we found a majority of PNGMP with neutralizing antibodies (NAb) to dengue and Zika viruses, with some NAb to chikungunya, Japanese encephalitis, Ross River, and Murray Valley encephalitis viruses. Sero-prevalence to Barmah Forest and West Nile viruses was less common.

**What is the impact on readiness and force health protection?**

This study shows the potential circulation of multiple mosquito-borne viruses in Papua New Guinea. The alarmingly high prevalence of arbovirus in tested Papua New Guinea military personnel reveals the significant risk of environmental exposure to mosquito-borne diseases in that country. These study findings indicate the threat posed to U.S. combatant commands operating in the region, illustrating the need for robust preventive measures that minimize bites from infected mosquitoes. Implementation of thorough vector control strategies and personal protective measures will be critical for mitigating existing arbovirus risks for personnel traveling to or exercising in Papua New Guinea.

Papua New Guinea, the world's third largest island country, is located in the Southwest Pacific, within the U.S. Indo-Pacific Command (INDOPACOM) area of responsibility. During World War II (WWII), Papua New Guinea was a crucial operational theater for the war in the Pacific, with a Japanese invasion in 1942 and subsequent Allied campaign—of primarily Australian and American forces—trying to expel the Japanese. A significant number of mosquito-borne pathogen infections were reported among active Allied troops during WWII after deployment to Papua New Guinea, ^1 , 4^ with DENV disease one of the major causes of morbidity among soldiers. ^5^

Recently, U.S. military presence in Papua New Guinea has increased, with several multi-national, joint exercises conducted in response to strategic pressures arising from the expansion of China's military presence in the Southwest Pacific. Surveillance conducted in Papua New Guinea in 2019 revealed a high prevalence rate of ZIKV and moderate CHIKV infections among Papua New Guinea military personnel (PNGMP) located at Manus Island and Wewak barracks. ^6 - 8^ ZIKV and CHIKV diseases are the second and third most significant arboviral disease threats to U.S. military, particularly during deployments to high-risk regions. ^5^ *Aedes* mosquitoes, the primary vector transmitting ZIKV, are found in nearly 200 U.S. military installations worldwide. From January 1, 2013 through December 31, 2022, 212 ZIKV cases were reported among U.S. service members, based on data from the Disease Reporting System internet and laboratory records from the Composite Healthcare System. ^9^ Meanwhile, CHIKV cases within the MHS are increasing, with a significant proportion of those infected experiencing long-term rheumatic complications. ^10^

Other arboviruses such as Ross River virus (RRV), Barmah Forest virus (BFV), Japanese encephalitis virus (JEV), and Murray Valley encephalitis virus (MVEV) infections have also been reported in Papua New Guinea. ^11 , 12^ RRV and BFV, both considered typical Australian arboviruses, are the leading causes of human arboviral diseases in that country, ^13^ which is less than 100 miles south of Papua New Guinea. JEV and MVEV are endemic in Papua New Guinea and northern Australia. ^14^ In 2016 and 2017, RRV and BFV outbreaks occurred among Australian Defence Force (ADF) personnel in the Shoalwater Bay Training Area of Queensland, in northern Australia. ^15 , 16^ A joint training exercise in 1997 resulted in at least 8 U.S. service members contracting RRV among approximately 9,000 U.S. marines and Australian soldiers participating in ground exercises in Queensland. ^17^ In 2022, a JEV outbreak in Australia resulted in 45 reported cases and 7 deaths. No new human cases of JEV were recorded in Australia from December 2022 until December 2024, when a JEV case was reported in Victoria, in southern Australia. Although the origin of the outbreak remains uncertain, it is believed that migratory birds or wind-blown mosquitoes may have introduced JEV from Papua New Guinea to Australia. ^18^

There are a few licensed vaccines currently available for arboviruses, and those that are available carry several caveats. The CHIKV vaccine, Ixchiq, approved by the U.S. Food and Drug Administration in 2023, is not yet widely licensed in other countries. For DENV, Dengvaxia (CYD-TDV) is approved in several countries but is only recommended for individuals ages 9-45 years with confirmed prior dengue infection. Due to the risk of severe disease in seronegative recipients, Dengvaxia is not ideal for broad public health use. In contrast, safe and effective vaccines that have long existed for JEV and yellow fever virus (YFV) are included in routine immunization programs in endemic regions.

For many other medically important arboviruses—including ZIKV, RRV, and MVEV—no licensed human vaccines currently exist, highlighting a significant gap in global prevention efforts. Furthermore, there are no specific anti-arboviral treatments for those diseases; clinical management primarily focuses on symptom relief.

Prevention of mosquito-borne arboviral diseases largely relies on personal protective measures, including long-sleeved uniforms, bed nets, permethrin treatment of uniforms and nets, and DEET (N,N-diethyl-meta-toluamide)-based mosquito repellents. Given the widespread presence of mosquito vectors in tropical and subtropical areas, arboviruses will likely continue to spread beyond their original regions of discovery, making force health protection in INDOPACOM increasingly challenging.

Use of antibody testing methods such as ELISA (enzyme-linked immunosorbent assay) for determining arbovirus exposure for surveillance is becoming a significant challenge due to broad cross-reactivity among the alphavirus and flavivirus families co-circulating in the same area. ^19^ Risk of co-infection further complicates serological interpretation.

Although a few serological studies—most conducted in the 1970s—have documented arbovirus exposure and outbreaks in Papua New Guinea, in addition to our own surveillance efforts in 2019, comprehensive epidemiological data remain limited due to lack of diagnostic capacity, inconsistent clinical case reporting, and weak surveillance infrastructure in much of Papua New Guinea. The COVID-19 pandemic placed additional strain on Papua New Guinea health systems. As a result, the true distribution, burden, and temporal trends of arboviral diseases in Papua New Guinea remain incompletely defined.

This study aims to address this important gap in data from Papua New Guinea by providing updated arbovirus serology data from PNGMP stationed at Lae Barracks on the country's eastern coast **(Figure 1)** . We measured neutralizing antibodies (NAb) against key arboviruses in this population. Our 2019 survey found high ZIKV and moderate RRV and CHIKV exposure at Wewak and Manus barracks. This follow-up study evaluates whether exposure patterns have shifted, especially post-COVID-19 pandemic.

**FIGURE 1.**
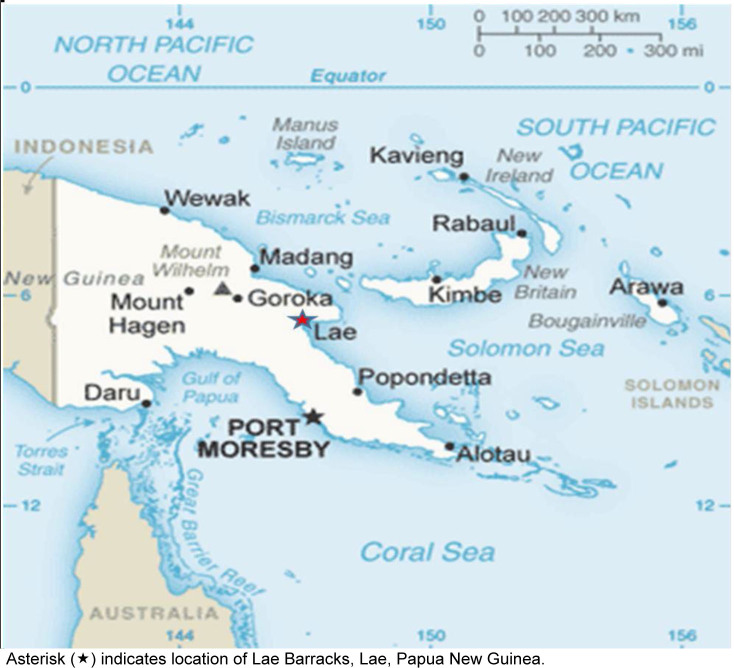
Location of Serological Survey of Papua New Guinea Military Personnel, 2023

## Methods

The study was approved by the Papua New Guinea Medical Research Advisory Committee (MRAC, no. 18-21) and the Department of Australian Defence and Veteran Affairs Human Research Ethics Committee (DDVA HREC, no. 084-18, no. 157-19). Written informed consent was obtained from all participants.

### Study Population Demographics

This study was part of an infectious disease surveillance program conducted by the ADF in conjunction with the Papua New Guinea Defence Force. A total of 185 PNGMP from Lae Barracks were recruited between April 20, 2023 and May 9, 2023. A convenience sample of serum specimens from PNGMP present at Lae Barracks during this period was collected. Participants provided written informed consent for additional infectious disease testing. Additional demographic and exposure data, including serum collection dates, age, sex, travel and vaccination history, and mosquito bite prevention measures, were self-reported via a study questionnaire. Due to security requirements, information on military occupational specialty, rank, and deployment history were not collected. The age range of participants was 24–60 years, with a median age of 35 years. The largest participant age group was 30-39 years (38.9%, n=72), followed by 20-29 years (27%, n=50). The cohort was predominantly male (96.2%, n=178). Forty-two participants reported domestic travel (22.7%), primarily to the capital, Port Moresby, or East or West Highlands regions. Ninetynine participants (53.5%) reported travel history to Australia, but only 11 (5.9%) had traveled to Australia within 3 months prior to blood collection. All participants were asymptomatic at the time of blood withdrawal, with no reported history of prior infectious disease infections and outcomes, and no reported prior vaccination for JEV and YFV. Demographic features and mosquito prevention practices reported by survey respondents are summarized in **Table 1** .

**TABLE 1.** Demographic Features and Mosquito Prevention Practices Reported for Papua New Guinea Military Study Participants

	No.	%
Total Blood Donors	185	100
Age, y		
20–29	50	27.0
30–39	72	38.9
40–49	25	13.5
>=50	38	20.5
Sex		
Male	178	96.2
Female	7	3.8
Travel		
Domestic	42	22.7
Overseas (3 months before blood withdrawal)	11	5.9
Australia	99	53.5
Mosquito prevention measures		
Mosquito coils	0	0.0
Screens	19	10.3
Mosquito bed net	98	53.0
House insecticide spray	108	58.4
Long sleeves	27	14.6
Combined	175	94.6
Vaccination against arboviruses (% of total)		
JEV and YFV	0	0.0

Abbreviations: y, years; JEV, Japanese encephalitis virus; YFV, yellow fever virus.

All serum samples were bar-coded, stored at -20°C and transported to Brisbane, Australia for neutralization analysis against the arbovirus strains as detailed in the **Supplementary Table** .

**SUPPLEMENTARY TABLE.** Arboviral Strains Used for Neutralization Assay in this Study

Name	Strain	Genbank No.
Barmah Forest virus	BH2193	NC_001786
Chikungunya virus	Reunion strain	DQ443544
Ross River virus	QML strain	GQ433354
Dengue virus 1	Hawaii	KM204119
Dengue virus 2	NGC	M29095.1
Dengue virus 3	H-87	M93130.1
Dengue virus 4	H-241	AY947539.1
Japanese encephalitis virus	Nakayama	EF571853.1
West Nile virus (Kunjin subtype)	MRM61C	KX394398.1
Murray Valley encephalitis virus	MK6684	KF751869

Abbreviation: No., number.

### Cells, Viruses and NAb Assays

Vero cells (African green monkey kidney epithelial cells) and C6 / 36 mosquito cells were routinely cultured in the laboratory and used for micro-neutralization assays. The arboviral strains used for NAb assays in this study are listed in the **Supplementary Table** . The virus stock preparation and serum NAb titers were assessed using a micro-neutralization assay and modified according to methods previously described. ^6 - 8^ NAb titer greater than or equal to 20 for flaviviruses and greater than or equal to 10 for alphaviruses were considered positive.

### Statistical Analysis

Data analysis was performed using GraphPad version 9.0 and an online Chisquare test calculator ( https://www.socscistatistics.com/tests/chisquare2/default2.aspx ) to compare the arbovirus sero-positivity proportions among different age groups. *P* -values less than or equal to 0.05 were considered statistically significant.

## Results

### Sero-Prevalence Determined by Pathogen-specific NAb

**FIGURE 2.**
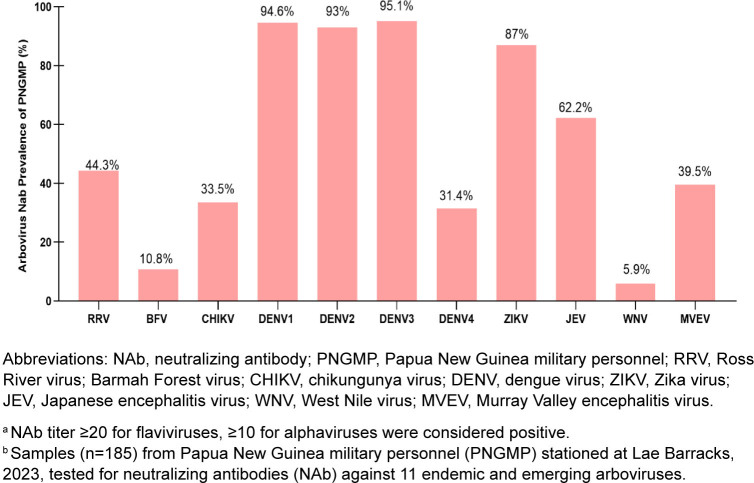
Arbovirus Sero-Prevalence ^a^ Among Papua New Guinea Military Personnel ^b^ Based on Neutralizing Antibody Assays, 2023

**FIGURE 3.**
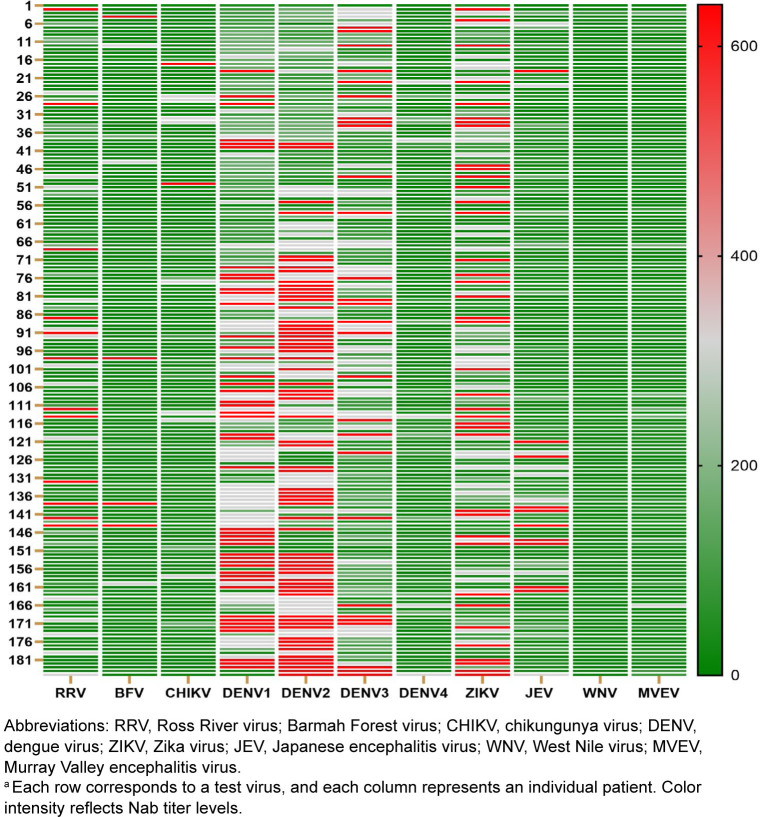
Heatmap Representation ^a^ of Neutralizing Antibody Titers in Papua New Guinea Military Personnel, 2023

**FIGURE 4.**
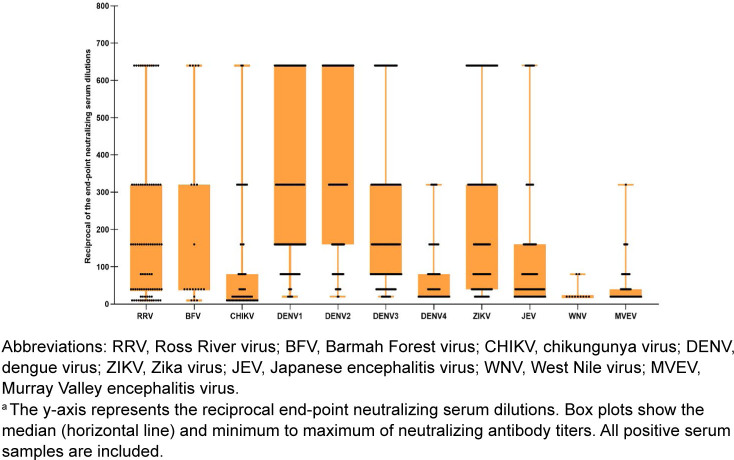
Box Plots ^a^ Illustrating Neutralizing Antibody Titers for 11 Arboviruses Tested in Papua New Guinea Military Personnel, 2023

As shown in **Table 2** and **Figures 2** – **4** , the 2023 PNGMP cohort exhibited very high sero-prevalence for DENV-1 (95%, n=175), DENV-2 (93%, n=172), and DENV-3 (95.1%, n=176), indicating widespread exposure or cross-reactivity NAbs among these serotypes. Mean NAb titers were also high for DENV-2–4, suggesting stronger or more frequent immune responses. Lower DENV-4 sero-prevalence (31%, n=57), with a significantly lower mean titer of 79.0, suggests that this serotype may be less commonly circulating or elicits a weaker immune response in this population. ZIKV was also prominent, with high sero-prevalence (87%, n=161) and a strong mean titer of 265.1, while JEV was also notable, with 62.2% (n=115) positivity and mean titer of 126.3. CHIKV (33.5%, n=62), RRV (44.3%, n=82), and MVEV (39.5%, n=73) showed moderate sero-positivity, indicating endemic but less dominant circulation. WNV (5.9%, n=11) and BFV (10.8%, n=20) demonstrated low sero-prevalence, suggesting either minimal exposure or low transmission rates in the study population. These findings reveal a high level of DENV and ZIKV exposure in PNGMP, with a still notable proportion exposed to CHIKV, RRV, JEV, and MVEV, and lower exposures to BFV and WNV.

**TABLE 2.** Arbovirus Neutralizing Antibody Positivity ^a^ Among Different Papua New Guinea Military Participants Before 2023

Virus		Total Positivity				Positivity by Age Group								
		All Samples (n=185)				20–29 (n=50)		30–39 (n=72)		40–49 (n=25)		>=50 (n=38)		Chi Square
		No.	%	Mean NAb Titer	95% CI	No.	%	No.	%	No.	%	No.	%	*p* -value
Flavivirus														
DENV group	DENV-1	175	94.6	303.9	271.0–336.8	46	92.0	68	94.4	24	96.0	37	97.4	0.998
	DENV-2	172	93.0	362.9	327.0–398.8	46	92.0	66	91.7	23	92.0	37	97.4	0.997
	DENV-3	176	95.1	226.5	195.8–257.2	46	92.0	67	93.1	25	100	38	100	0.122
	DENV-4	57	31.4	79.0	55.7–102.2	12	24.0	26	36.1	8	32.0	12	31.6	0.782
JEV group	JEV	115	62.2	126.3	93.4–159.2	27	54.0	48	66.7	17	68.0	23	60.5	0.9
	WNV	11	5.9	30.9	14.6–47.2	2	4.0	4	5.6	0	0.0	5	13.2	N.D
	MVEV	73	39.5	46.6	34.9–58.3	22	44.0	26	36.1	9	36.0	15	39.5	0.944
Spondweni group	ZIKV	161	87.0	265.1	228.2–301.9	41	82.0	62	86.1	24	96.0	34	89.5	0.215
Alphavirus														
	RRV	82	44.3	193.9	146.8–241.0	18	36.0	30	41.7	14	56.0	20	52.6	0.673
	CHIKV	62	33.5	89.2	52.5–125.9	15	30.0	20	27.8	14	56.0	13	34.2	0.373
	BFV	20	10.8	202.5	86.6–318.4	4	8.0	7	9.7	1	4.0	8	21.1	N.D.

Abbreviations: n, number; No., number; NAb, Neutralizing antibody; CI, confidence interval; DENV, dengue virus; JEV, Japanese encephalitis virus; WNV, West Nile virus; N.D., not done; MVEV, Murray Valley encephalitis virus; ZIKV, Zika virus; RRV, Ross River virus; CHIKV, chikungunya virus; BFV, Bahmah Forest virus.

a NAb titer >=20 was considered ‘positive’ for flaviviruses; NAb titer >=10 was considered ‘positive’ for alphaviruses.

In a Chi-square analysis, the sero-prevalence of RRV, CHIKV, DENV1-4, ZIKV, MVEV, and JEV did not significantly differ by age group **(Table 2)** . Due to the low sero-prevalence of BFV and WNV, NAb sero-positivity rates were not compared by age groups. Sex-stratified analysis was not conducted due to the predominantly male study population.

### Multiple NAb Positivity Among Alphavirus

Among alphaviruses, dual or multiple NAb sero-positivity patterns were observed: BFV ^+^ / CHIKV ^+^ in 3 cases (1.6%), BFV ^+^ / RRV ^+^ in 5 cases (2.7%), CHIKV ^+^ / RRV ^+^ in 34 cases (18.3%), and triple positivity BFV ^+^ / CHIKV ^+^ / RRV ^+^ in 7 cases (3.8%) (data not shown).

### Multiple NAb Positivity Among Dengue Virus and Flavivirus

As shown in **Table 3** , nearly one-third (28.1%) of PNGMP in 2023 had pan-sero-type immunity, or positivity to all 4 DENV serotypes. Moreover, 64.3% of participants were positive to 3 serotypes, suggesting multiple past infections or strong cross-reactive responses, likely due to co-circulation or sequential infections with different serotypes. Only a small fraction (5.4%) was positive to only 1 or 2 serotypes, and 2.2% were negative for all 4, indicating minimal or no prior DENV exposure in this subset.

**TABLE 3.** Dengue Virus Neutralizing Antibody Multi-Seropositivity Among 185 Participating Papua New Guinea Military Personnel, 2023

Number of Positive DENV Serotypes	Number of Participants	
	No.	%
Positive to all 4 serotypes	52	28.1
Positive to 3 serotypes	119	64.3
Positive to 2 serotypes	6	3.2
Positive to 1 serotype	4	2.2
Positive to at least 1 serotype	181	97.8
Negative to all serotypes	4	2.2

Abbreviations: DENV, dengue virus; No., number.

A significant majority (92.9%) of study participants were sero-positive to 4 or more flaviviruses **(Table 4)** , indicating an extensive exposure or high cross-reactive antibody responses. The largest groups consisted of individuals positive to 6 (31.4%) or 5 (30.3%) flaviviruses, reflecting multi-flavivirus circulation or repeated exposures. Only a small minority (5.9%) were positive to 3 or fewer flaviviruses, suggesting limited exposure in only this small portion of the participant population. Notably, 2 individuals (1.1%) were sero-positive to all 8 flaviviruses tested, suggesting either uncommonly broad exposure histories or high cross-reactivity of their serum antibodies.

**TABLE 4.** Multi-Seropositivity to Flaviviruses Among Participating Papua New Guinea Military Personnel (n=185), 2023

Number of Positive Flaviviruses	Number of Participants	
	No.	%
Positive to 8 flaviviruses	2	1.1
Positive to 7 flaviviruses	17	9.2
Positive to 6 flaviviruses	58	31.4
Positive to 5 flaviviruses	56	30.3
Positive to 4 flaviviruses	39	21.1
Positive to 3 flaviviruses	5	2.7
Positive to 2 flaviviruses	1	0.5
Positive to 1 flavivirus	5	2.7
Positive to 0 flaviviruses	2	1.1

Abbreviation: no., number.

These data indicate a high burden of arbovirus transmission or immunological imprinting in the region, consistent with the co-endemicity of multiple flaviviruses and alphaviruses such as DENV, ZIKV, RRV, CHIKV, JEV, MVEV, and WNV.

## Discussion

Arboviral infections represent substantial and ongoing threats to the operational readiness of U.S. military personnel deployed to endemic regions such as Papua New Guinea. These results, demonstrating high arbovirus sero-positivity and co-positivity rates among PNGMP in 2023, reaffirm that Papua New Guinea is a highly endemic area for multiple arboviruses. These findings have important implications for U.S. forces deployed under the recently expanded defense cooperation agreements between the U.S., Australia, and Papua New Guinea.

At the Lae Barracks in 2023, PNGMP exhibited extremely high DENV sero-positivity, with 98% of participants demonstrating NAb reactivity to at least 1 DENV serotype. The most frequently co-detected serotypes were DENV-1, DENV-2, and DENV-3, with DENV-4 showing the lowest prevalence. This distribution aligns with historical data indicating the persistent endemic circulation of DENV-1–3 in PNG and lower levels of DENV-4 introduction into Australia from Papua New Guinea from 1999 through 2020. ^20 - 22^

Accurate interpretation of DENV NAb results in hyper-endemic regions such as Papua New Guinea is complicated by repeated exposures to multiple DENV serotypes. Primary DENV infection provides life-long immunity to the infecting serotype virus but only transient, partial protection against the other 3 serotypes. ^23^ Secondary infections often result in broad, cross-reactive NAb responses that can persist long-term. ^24^ Moreover, other endemic flaviviruses in Papua New Guinea, including ZIKV, JEV, WNV, and MVEV, can also cross-react with DENV in NAb assays, further complicating serological interpretation. ^25^ Therefore, it is difficult to determine true viral exposure using the traditional greater than or equal to (>=) 4-fold NAb titer difference between real infection virus and its cross-reaction virus, as many PNGMP samples exhibited high NAb titers against multiple DENV serotypes and other flavivirus, mainly ZIKV.

Nevertheless, the high sero-positivity strongly suggests the ongoing co-circulation and repeated DENV exposure in this population. The risk of secondary DENV infections, which are associated with more severe disease through antibody-dependent enhancement (ADE), remains a concern in populations with high prior exposure. The apparent lack of reported severe dengue cases among PNGMP, however, may indicate that a substantial proportion of infections are asymptomatic or present only with mild, non-specific symptoms. Asymptomatic or sub-clinical infections represent the majority of DENV infections, particularly in endemic settings, and under-reporting or mis-classification of mild cases as malaria or undifferentiated febrile illness may also contribute to this observation. These findings underscore the importance of paired serological and clinical surveillance to fully understand the burden of disease and guide appropriate preventive strategies in PNGMP. ^26^

High ZIKV Nab sero-positivity (87%) among PNGMP 2023 at Lae Barracks shows no statistically significant difference compared to the 2019 survey of PNGMP (65%) at Wewak and Manus Island barracks prior to the COVID-19 pandemic. This prevalence aligns with the high level of ZIKV sero-prevalence (49-63%) in Polynesia, which includes endemic regions, ^27^ and is significantly higher than the 15.6% average reported across the broader Western Pacific. ^28^ The elevated prevalence among PNGMP likely reflects increased exposure risks due to greater contact with mosquito habitats during field operations, higher endemicity in deployment locations, or insufficient use of personal protective measures. This underscores the need for targeted vector control, health education, and operational adjustments to reduce transmission risk and protect force health.

Despite widespread ZIKV exposure among PNGMP, our literature review found no reports of congenital ZIKV syndrome (CZS) in Papua New Guinea, possibly due to limited diagnostic capabilities and under-reporting in the country. Additionally, time of exposure may play an important role, as the highest risk of CZS occurs in the first trimester of pregnancy. Cross-immunological protection afforded by high levels of DENV immunity has also been identified as a contributor to reduce CZS development. ^29 , 30^ The predominantly young adult male PNGMP cohort may not reflect exposure or infection rates among pregnant women, who are at greatest risk for CZS.

Similarly, CHIKV NAb sero-positivity (33.5%) among PNGMP mirrors rates observed in 2019, suggesting sustained, low level CHIKV endemicity following the 2012 Papua New Guinea outbreak. Cases of CHIKV imported to Australia from Papua New Guinea from 2016 to 2020 evidence Papua New Guinea's role as a persistent source of CHIKV transmission risk. ^8 , 31 , 32^

Sero-prevalence data also confirm ongoing exposure of PNGMP to other arboviruses historically reported in Papua New Guinea—including RRV, BFV, JEV, WNV, and MVEV—with no significant variation by age, reflecting sustained transmission or early life exposure.

A key strength of this study is the use of the gold standard NAb assay to detect virusspecific antibodies, which correlate strongly with immune protection. While NAb assays cannot distinguish antibody isotypes (IgM vs. IgG) ^33^ and may retain low level cross-reactivity among related arboviruses, they remain the most reliable serological method. Cross-neutralizing antibodies between DENV and ZIKV are widely documented, with repeated DENV-exposed individuals often exhibiting stronger and more durable cross-neutralizing responses to ZIKV. Similarly, ZIKV infection can induce cross-neutralizing antibodies against DENV, but the extent and duration of this effect can vary. ^25^ To minimize false positives from cross-reactivity, we applied a conservative sero-positivity limit of greater than or equal to (>=) 1:20 NAb titer for flaviviruses, although precise exposure determination remains challenging in hyper-endemic settings like Papua New Guinea.

These findings have critical implications for U.S. military personnel deployed in Papua New Guinea. Personnel without prior exposure or natural immunity face heightened risks of infection and severe disease, particularly for arboviruses such as DENV, ZIKV, and CHIKV. Asymptomatic or mildly symptomatic infected personnel could inadvertently export arboviruses to other regions with competent vector populations, echoing the past global ZIKV and CHIKV outbreaks.

Given these risks, continuous arboviral and clinical surveillance among military and local populations is essential for detecting symptomatic and asymptomatic infections. Surveillance should also be complemented by entomological monitoring to track spatio-temporal changes in mosquito populations, including species diversity, vector competence, biting behavior, longevity, and dispersal capacity. Environmental factors such as rainfall and temperature, which influence mosquito breeding and activity, as well as zoonotic and sylvatic surveillance, for spillover risk detection, must be integrated for comprehensive risk assessment.

Expanded studies with larger, more demographically diverse cohorts and application of more specific serological assays (e.g., epitope-based ELISAs) will further clarify exposure patterns and guide force health protection measures. Although this study focused on PNGMP, it provides a valuable insight into the endemic risk landscape facing deployed U.S. forces. The endemic and emerging arboviruses circulating in Papua New Guinea pose a sustained threat to U.S. military readiness in this strategically important region. Proactive surveillance, force health education, vector control strategies, and vaccination will be essential to mitigate the risks and safeguard deployed personnel.
